# Acute Psychosis as the Sole Initial Manifestation of Systemic Lupus Erythematosus: A Diagnostic Challenge

**DOI:** 10.7759/cureus.102002

**Published:** 2026-01-21

**Authors:** Airenakho Emorinken, Patrick O Adunbiola, Mercy O Dic-Ijiewere, Mojeed O Rafiu, Ndidi N Akerele

**Affiliations:** 1 Rheumatology Unit, Department of Internal Medicine, Irrua Specialist Teaching Hospital, Irrua, NGA; 2 Department of Medicine, Ambrose Alli University, Ekpoma, NGA; 3 Endocrinology Unit, Department of Internal Medicine, Irrua Specialist Teaching Hospital, Irrua, NGA; 4 Cardiology Unit, Department of Internal Medicine, Irrua Specialist Teaching Hospital, Irrua, NGA; 5 Nephrology Unit, Department of Internal Medicine, Irrua Specialist Teaching Hospital, Irrua, NGA

**Keywords:** autoimmune psychosis, first-episode psychosis, immunosuppressive therapy, lupus psychosis, neuropsychiatric systemic lupus erythematosus (npsle), systemic lupus erythromatosus

## Abstract

Neuropsychiatric systemic lupus erythematosus (NPSLE) represents one of the most complex and heterogeneous manifestations of systemic lupus erythematosus (SLE). Psychosis is an uncommon but severe feature of NPSLE and usually occurs in the setting of established multisystem disease. Acute psychotic presentation as the sole initial manifestation of SLE is exceptionally rare and poses considerable diagnostic challenges. We report the case of a 24-year-old woman with no prior psychiatric or medical history who developed abrupt-onset psychosis characterized by insomnia, aggression, persecutory delusions, and auditory hallucinations. Initial evaluation suggested first-episode psychosis; however, poor response to antipsychotic therapy and the presence of unexplained pancytopenia with elevated inflammatory markers prompted further investigation. Autoimmune testing revealed high-titer antinuclear antibodies, elevated anti-double-stranded DNA, anti-Smith, and anti-ribosomal P antibodies, hypocomplementemia, and a positive direct Coombs test. Infectious, metabolic, toxic, and structural neurological causes were systematically excluded. The patient fulfilled the 2019 European Alliance of Associations for Rheumatology/American College of Rheumatology classification criteria for SLE and met diagnostic attribution for lupus psychosis. Treatment with high-dose pulse intravenous methylprednisolone and intravenous cyclophosphamide resulted in rapid and sustained resolution of psychotic symptoms, with normalization of laboratory abnormalities. Acute psychosis may rarely be the first and isolated manifestation of SLE. This case highlights the importance of considering NPSLE in young patients presenting with first-episode psychosis and underscores the role of early immunosuppressive therapy in achieving favorable outcomes, even in resource-limited settings.

## Introduction

Systemic lupus erythematosus (SLE) is a chronic multisystem autoimmune disease characterized by immune dysregulation and autoantibody production, with the potential to involve multiple organ systems, including the central nervous system [1]. Its clinical course is highly heterogeneous, ranging from mild mucocutaneous and musculoskeletal manifestations to severe, life-threatening organ involvement. Neuropsychiatric manifestations represent some of the most challenging aspects of SLE due to their diverse presentations, complex pathophysiology, and significant impact on morbidity and quality of life [2,3].

Neuropsychiatric systemic lupus erythematosus (NPSLE) comprises a broad spectrum of neurological and psychiatric syndromes involving the central and peripheral nervous systems. These include cognitive dysfunction, mood and anxiety disorders, seizures, cerebrovascular disease, movement disorders, and psychosis [4]. Neuropsychiatric symptoms may occur at any stage of SLE but are reported in 39-50% of patients during the course of the disease [2,5]. However, prevalence estimates vary widely from 12% to 95% due to differences in study design, attribution models, and population characteristics [2,6]. Lupus psychosis is classified among the diffuse neuropsychiatric syndromes of SLE and represents one of the less common but clinically significant manifestations of NPSLE [4,5].

Psychosis is an uncommon but severe manifestation of NPSLE, occurring in about 0.6-11% of patients [3,5]. It typically presents early in the disease course and is usually associated with other clinical or serological features of active systemic disease [5,7]. Acute psychosis presenting as the sole initial manifestation of SLE is exceedingly rare and represents a major diagnostic challenge. Such presentations frequently mimic primary psychiatric disorders, increasing the risk of misdiagnosis or delayed recognition of an underlying autoimmune etiology [8-10]. The differential diagnosis of acute psychosis in a young adult includes primary psychiatric disorders, substance-induced psychosis, central nervous system infections, metabolic encephalopathies, autoimmune encephalitis, and structural brain lesions [3,6]. Neuroimaging, particularly magnetic resonance imaging (MRI), plays an important role in excluding these mimics and identifying inflammatory or vasculopathic changes that may support an autoimmune origin [5,6].

The pathogenesis of NPSLE remains incompletely understood. Current evidence supports a predominant role for immune-mediated mechanisms targeting the central nervous system, which may occur independently of overt systemic disease activity [2,11]. The involvement of multiple, overlapping pathogenic pathways contributes to the marked heterogeneity of NPSLE and complicates diagnostic attribution [12]. Among the proposed mechanisms, autoantibody-mediated neuronal dysfunction has been most extensively studied. In particular, anti-ribosomal P and anti-N-methyl-D-aspartate receptor antibodies have been implicated in both experimental models and clinical studies, especially in relation to psychiatric and cognitive manifestations of NPSLE [2,11].

In 1999, the American College of Rheumatology (ACR) proposed standardized case definitions for neuropsychiatric syndromes associated with SLE to aid clinical classification and research [13]. Despite this framework, no validated diagnostic criteria exist for NPSLE, and diagnosis relies on expert clinical judgment, careful attribution, and exclusion of alternative causes such as primary psychiatric disorders, substance-induced psychosis, infections, metabolic encephalopathies, and other neurological conditions [14,15].

We report an unusual case of acute psychosis as the sole initial manifestation of SLE in a 24-year-old woman managed in a resource-limited setting, highlighting the diagnostic reasoning, therapeutic approach, and clinical outcome.

## Case presentation

A 24-year-old female university student with no previous medical or psychiatric history presented with a five-day history of abrupt behavioral change characterized by severe insomnia, irritability, aggression, and marked suspiciousness. She was observed responding to auditory hallucinations and expressed fixed persecutory delusions, believing that her classmates were conspiring to poison her. There was no history of substance use, recent infection, head trauma, travel, or exposure to prescription or illicit medications. She denied recent psychosocial stressors, and there was no family history of psychiatric illness. There were no antecedent symptoms suggestive of systemic disease, including fever, rash, headache, arthralgia, vomiting, seizures, or altered level of consciousness. Prior to the onset of symptoms, she had been academically stable and socially well-adjusted.

On physical examination, she was afebrile and appeared mildly pale. Vital signs were stable, with a blood pressure of 112/74 mmHg, heart rate of 98 beats per minute, respiratory rate of 18 breaths per minute, and oxygen saturation of 99% on room air. She was conscious and alert, oriented to person and place, but intermittently disoriented to time. Mental state examination revealed an anxious and labile affect, disorganized thought processes, second-person auditory hallucinations, and fixed persecutory delusions. She appeared disheveled and distracted. There were no focal neurological deficits, meningeal signs, or extrapyramidal features. The remainder of the systemic examination was unremarkable, with no cutaneous rashes, oral ulcers, alopecia, or clinical evidence of inflammatory arthritis.

She was admitted with a provisional diagnosis of first-episode psychosis and treated with intramuscular haloperidol 5 mg and promethazine 25 mg, followed by oral risperidone 2 mg daily.

Initial investigations demonstrated pancytopenia and elevated inflammatory markers, with otherwise normal renal, hepatic, metabolic, and thyroid profiles. Urine toxicology screening was negative for cannabis, cocaine, amphetamines, benzodiazepines, and opioids. Initial infectious screening, including blood culture and tests for malaria, human immunodeficiency virus, hepatitis B and C, and syphilis, was non-reactive.

Despite antipsychotic therapy, the patient remained psychotic and disorganized. In view of the poor clinical response and the presence of unexplained pancytopenia and elevated inflammatory markers, further evaluation was undertaken. Non-contrast computed tomography of the brain revealed no structural abnormalities. The radiology report noted no intracranial hemorrhage, mass effect, midline shift, hydrocephalus, or focal infarct. Ventricular and sulcal patterns were within normal limits. MRI could not be performed due to unavailability and financial constraints. Electroencephalography, transthoracic echocardiography, abdominal ultrasonography, and chest radiography were unremarkable. The persistence of psychotic symptoms in the context of atypical laboratory findings prompted a rheumatology consultation and subsequent autoimmune evaluation.

Serological testing revealed a high titer antinuclear antibody (ANA), elevated anti-double-stranded DNA (anti-dsDNA), anti-Smith (anti-Sm), and anti-ribosomal P antibodies, with hypocomplementemia. The direct Coombs test was positive, supporting immune-mediated hematologic involvement. Abnormal laboratory findings supporting the diagnosis of SLE are shown in Table 1.

**Table 1:** Abnormal laboratory findings supporting the diagnosis of SLE DNA: deoxyribonucleic acid; SLE: systemic lupus erythematosus; mg/dL: milligram per deciliter; g/dL: gram per deciliter; L: Liter; IU/mL: international unit per milliliter; U/mL: unit per milliliter; mg/L: milligram per liter; mm/h: millimeter per hour.

Investigation domain	Test	Result	Reference range
Hematology	Hemoglobin (g/dL)	9.4	12-16
	White blood cell count (×10⁹/L)	2.9	4.0-11.0
	Platelet count (×10⁹/L)	115	150-400
Inflammatory markers	Erythrocyte sedimentation rate (mm/h)	80	0-20
	C-reactive protein (mg/L)	10	<5
Autoimmune serology	Antinuclear antibody	1:2560 (homogeneous)	<1:80 (negative)
	Anti–double-stranded DNA (IU/mL)	155	<25
	Anti-Smith antibody	Positive	Negative
	Anti-ribosomal P antibody (U/mL)	68	<20
Complement levels	Complement C3 (mg/dL)	68	90-180
	Complement C4 (mg/dL)	7	10-40
Immunohematology	Direct Coombs test	Positive	Negative

Based on the presentation of acute psychosis, evidence of systemic inflammation and immune-mediated pancytopenia, characteristic immunologic abnormalities, and exclusion of infectious, metabolic, toxic, and structural neurological causes, a diagnosis of NPSLE presenting as lupus psychosis was established. The patient fulfilled the 2019 European Alliance of Associations for Rheumatology/American College Rheumatology classification criteria for SLE with a total score of 17 points, exceeding the diagnostic threshold of 10 points.

She received intravenous pulse methylprednisolone 1 g daily for three consecutive days, followed by oral prednisolone at 1 mg/kg/day with gradual tapering. Intravenous cyclophosphamide 500 mg every two weeks was initiated as induction therapy, planned for six doses. Hydroxychloroquine 200 mg twice daily was commenced, and risperidone 2 mg daily was continued for short-term symptom control.

**Figure 1:**
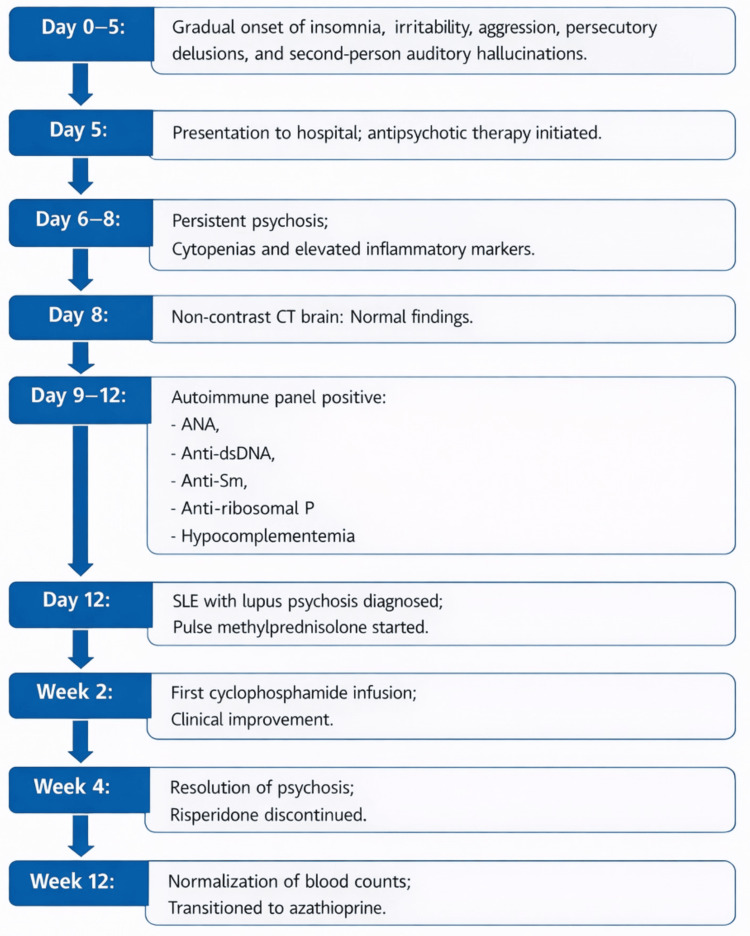
Timeline of key clinical events in this patient with lupus psychosis This timeline summarizes symptom onset, diagnostic workup, and treatment response in a patient with lupus psychosis. ANA: antinuclear antibody; Anti-dsDNA: anti–double-stranded DNA; Anti-ribosomal P: anti-ribosomal P antibody; NPSLE: neuropsychiatric systemic lupus erythematosus; HCQ: hydroxychloroquine.

Marked clinical improvement was observed following initiation of immunosuppressive therapy, with a substantial reduction in psychotic symptoms after pulse corticosteroids and further improvement after the first dose of cyclophosphamide. She was discharged on an oral steroid taper with plans for continued outpatient cyclophosphamide infusions. At two-week follow-up, hallucinations were less intrusive, and sleep had normalized, allowing gradual tapering and discontinuation of risperidone. By four weeks, she was asymptomatic, fully oriented, socially engaged, and demonstrated good insight. By 12 weeks, hemoglobin, white blood cell count, and platelet count had normalized, with corresponding declines in erythrocyte sedimentation rate and C-reactive protein (Table 2). She remained clinically stable, with no recurrence of psychosis or other neuropsychiatric manifestations. She was subsequently transitioned to oral azathioprine 100 mg daily for maintenance therapy, alongside hydroxychloroquine and low-dose prednisolone (10 mg daily). She continues regular follow-up with sustained clinical improvement. Figure 1 provides a structured visual timeline of the patient’s clinical course from onset to recovery.

**Table 2:** Hematologic and inflammatory parameters at baseline and 12 weeks Baseline values correspond to Days 5-7 (time of diagnostic evaluation). Follow-up values were obtained at Week 12 after initiation of immunosuppressive therapy. ESR: erythrocyte sedimentation rate; CRP: C-reactive protein; mm/h: millimeter per hour; g/dL: gram per deciliter; mg/L: milligram per liter; L: liter.

Parameter	Baseline	12 weeks	Reference range
Hemoglobin (g/dL)	9.4	11.6	12-16
White blood cell count (×10⁹/L)	2.9	5.0	4.0-11.0
Neutrophils (×10⁹/L)	1.6	2.8	2.0-7.5
Lymphocytes (×10⁹/L)	1.1	1.9	1.0-4.0
Platelet count (×10⁹/L)	115	196	150-400
ESR (mm/h)	80	22	<20
CRP (mg/L)	10	4	<5

## Discussion

Acute psychosis as the sole initial manifestation of SLE is exceptionally rare and poses significant diagnostic challenges. Although neuropsychiatric involvement occurs in a substantial proportion of patients with SLE during the disease course, psychosis remains one of its least frequent manifestations and rarely presents in isolation [2,4,5]. This case illustrates an uncommon presentation of NPSLE in which acute psychosis preceded any overt systemic features, underscoring the protean nature of SLE and the need for a high index of suspicion in young patients presenting with first-episode psychosis.

Psychosis in SLE typically occurs early in the disease course and is most often observed in the context of multisystem disease activity [5,7]. While previous studies have reported psychosis as an initial manifestation in a subset of patients, such cases almost invariably occur in the context of multisystem disease involvement [7,8]. In one cohort, 60% of patients presented initially with psychosis, all in the setting of concomitant multisystem lupus activity [7]. Presentation as an isolated neuropsychiatric syndrome, as seen in this patient, is rare and increases the risk of misdiagnosis as a primary psychiatric disorder, potentially delaying initiation of appropriate immunosuppressive therapy [3,9]. The absence of classical features such as rash, arthritis, serositis, or nephritis at presentation contributed to the diagnostic complexity and mirrors challenges described in earlier reports where NPSLE masqueraded as primary psychosis [9,10].

The clinical features observed, including hallucinations, persecutory delusions, disorganized thought processes, and behavioral disturbance, are consistent with lupus psychosis [3]. These manifestations reflect immune-mediated central nervous system injury and neuroinflammatory processes rather than a primary psychiatric disorder. However, potential overlap with medication-related effects, particularly corticosteroids, must be carefully considered [3,16]. Steroid-induced psychosis was confidently excluded, as psychiatric symptoms preceded corticosteroid exposure, and established risk factors such as lupus nephritis and hypoalbuminemia were absent [16].

The pathogenesis of NPSLE is multifactorial and includes autoantibody-mediated neuronal effects, cytokine-driven inflammation, immune complex deposition, microvascular injury, and disruption of the blood-brain barrier [2,4,11]. In this patient, high-titer ANA, elevated anti-dsDNA and anti-Sm antibodies, hypocomplementemia, and anti-ribosomal P antibody positivity supported an immune-mediated etiology. Although the sensitivity and specificity of anti-ribosomal P antibodies remain limited, their association with lupus psychosis is well documented and provides important supportive evidence in this context [5,17].

Evaluation of suspected NPSLE requires meticulous exclusion of infectious, metabolic, toxic, structural, and primary psychiatric causes [3,14]. MRI is the preferred neuroimaging modality for detecting subtle inflammatory or vasculopathic changes in NPSLE [6,18]. However, normal MRI findings do not exclude active disease, as a significant proportion of patients with clinically active NPSLE have unremarkable imaging [6,18]. In this case, MRI was unavailable due to resource constraints, highlighting challenges frequently encountered in low-resource settings. The absence of advanced neurodiagnostic testing represents a limitation, and while the clinical and serologic data strongly support lupus psychosis, definitive exclusion of other inflammatory or autoimmune encephalitides cannot be fully established.

Management of lupus psychosis focuses on controlling the underlying immune-mediated inflammatory process. High-dose corticosteroids remain the cornerstone of therapy, with cyclophosphamide commonly employed for induction in severe disease [5,16]. Maintenance therapy with agents such as azathioprine or mycophenolate mofetil is often required. In selected cases, biologics such as rituximab have been reported with some success in patients with refractory NPSLE [15,16]. The use of intravenous immunoglobulin and plasma exchange has also been described in refractory cases [2,14,15]. Adjunctive antipsychotic medications may assist with short-term symptom control but are insufficient as monotherapy. The rapid and sustained improvement observed in this patient after pulse methylprednisolone and cyclophosphamide provides strong inferential evidence supporting an autoimmune inflammatory mechanism [8,9].

This case highlights several important clinical lessons. Acute psychosis can be the initial and predominant manifestation of SLE, even in the absence of classical systemic features. In resource-limited settings where advanced neuroimaging is unavailable, careful clinical assessment, comprehensive serological evaluation, and systematic exclusion of alternative diagnoses can still achieve diagnostic certainty. Early recognition and timely initiation of immunosuppressive therapy are critical for favorable outcomes and may prevent unnecessary delays associated with prolonged psychiatric management alone. Nevertheless, the interpretation of this single case should remain cautious, acknowledging the inherent diagnostic limitations while emphasizing the clinical relevance of the presentation.

## Conclusions

Acute psychosis can be an uncommon initial presentation of SLE. This case highlights the importance of considering NPSLE in young adults with first-episode psychosis, especially when no prior psychiatric history is present. A structured approach focusing on exclusion of common mimics and timely serologic testing is essential for accurate diagnosis. Early initiation of immunosuppressive therapy can lead to marked improvement, even in resource-limited settings. This patient’s rapid response to treatment underscores the need to maintain a broad differential and to consider NPSLE in atypical or unexplained psychiatric presentations.
